# Supplementary figures and images for: AI-driven quantification of ground glass opacities in lungs of COVID-19 patients using 3D computed tomography imaging
Source: PLoS One. 2022 Mar 14;17(3):e0263916. doi: 10.1371/journal.pone.0263916 (PMC8920286; doi:10.1371/journal.pone.0263916)

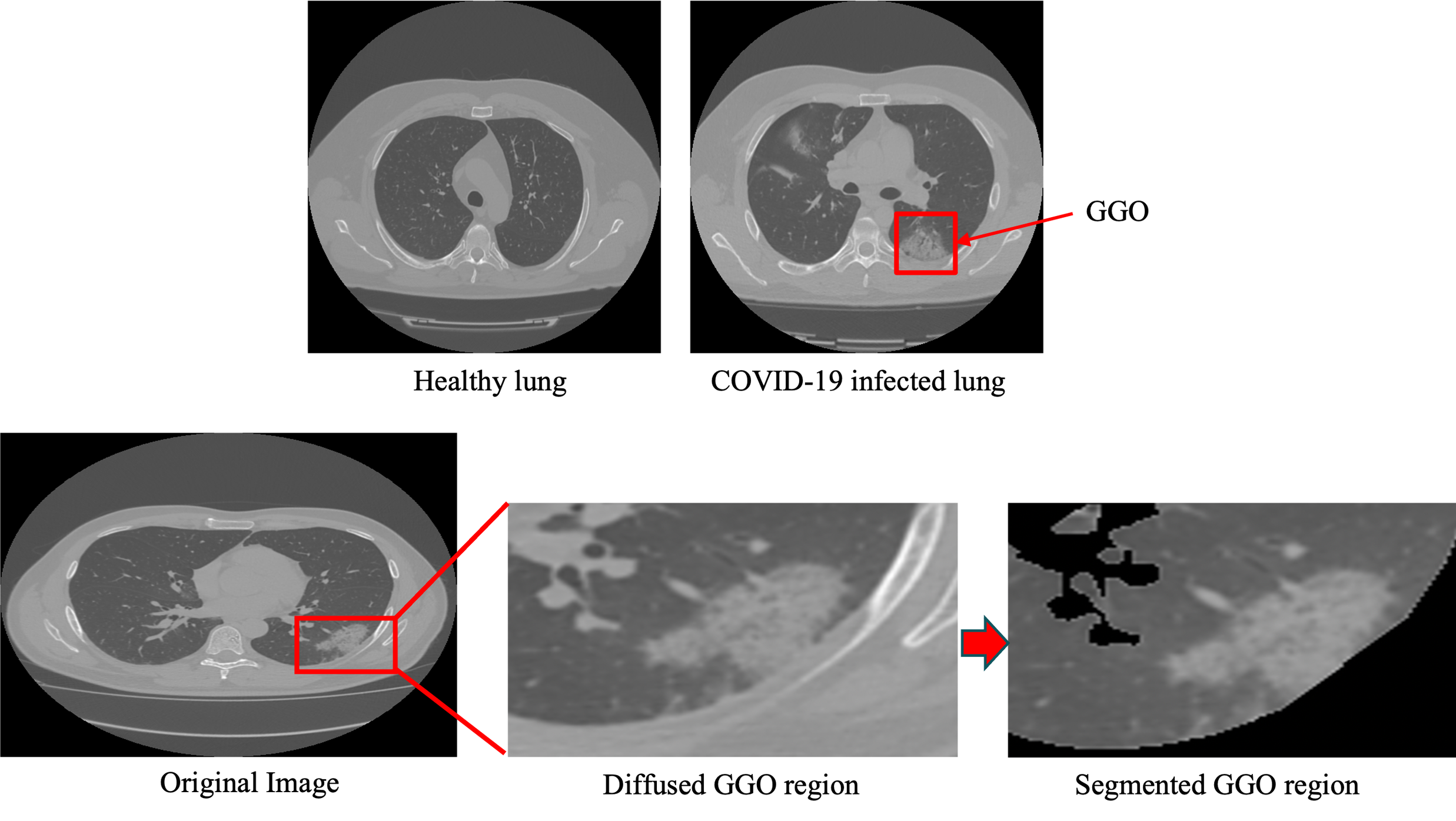

Supplement: S1 File — (ZIP) [file pone.0263916.s001.zip › Figure7.tif]

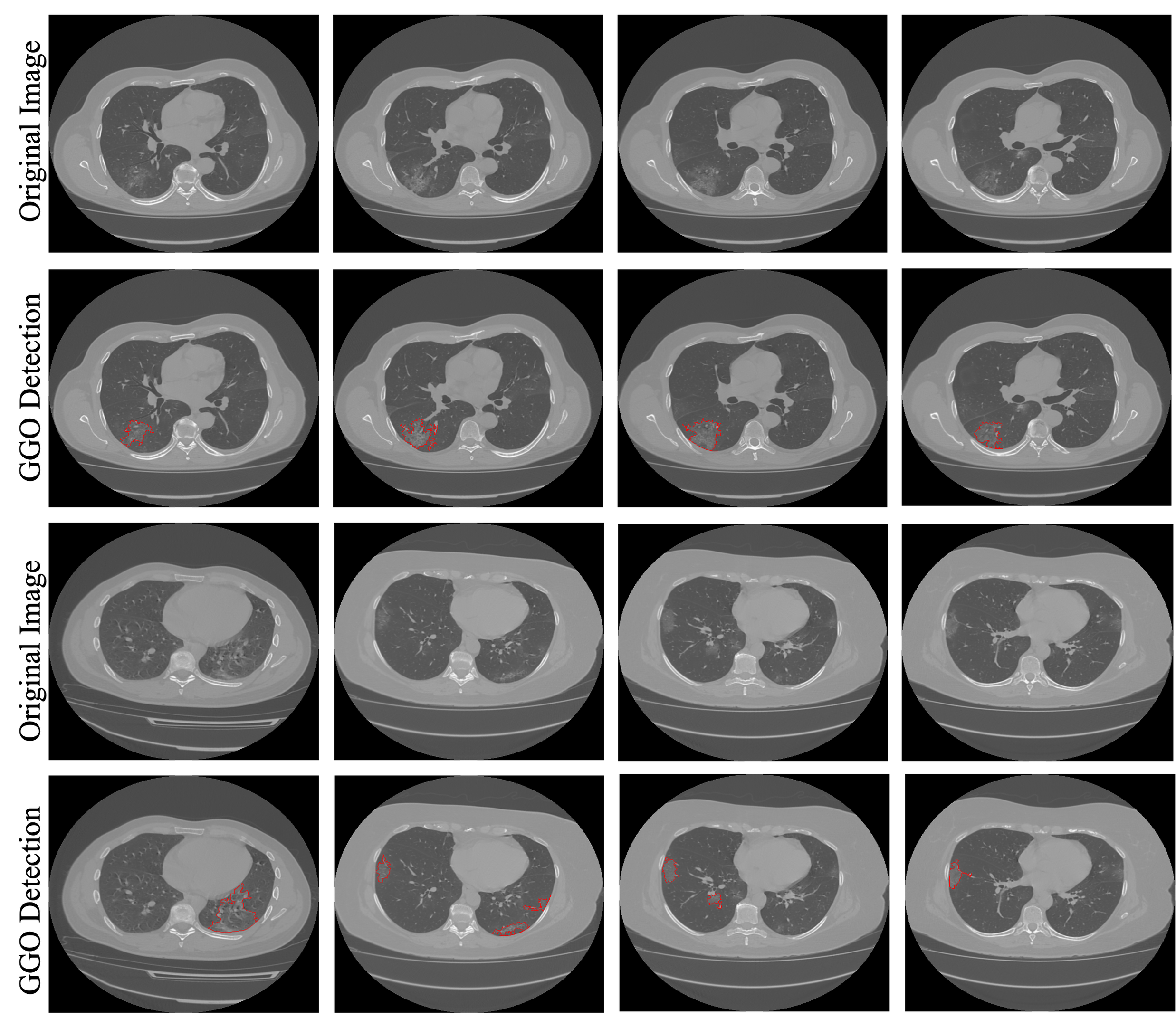

Supplement: S1 File — (ZIP) [file pone.0263916.s001.zip › Figure8.tif]

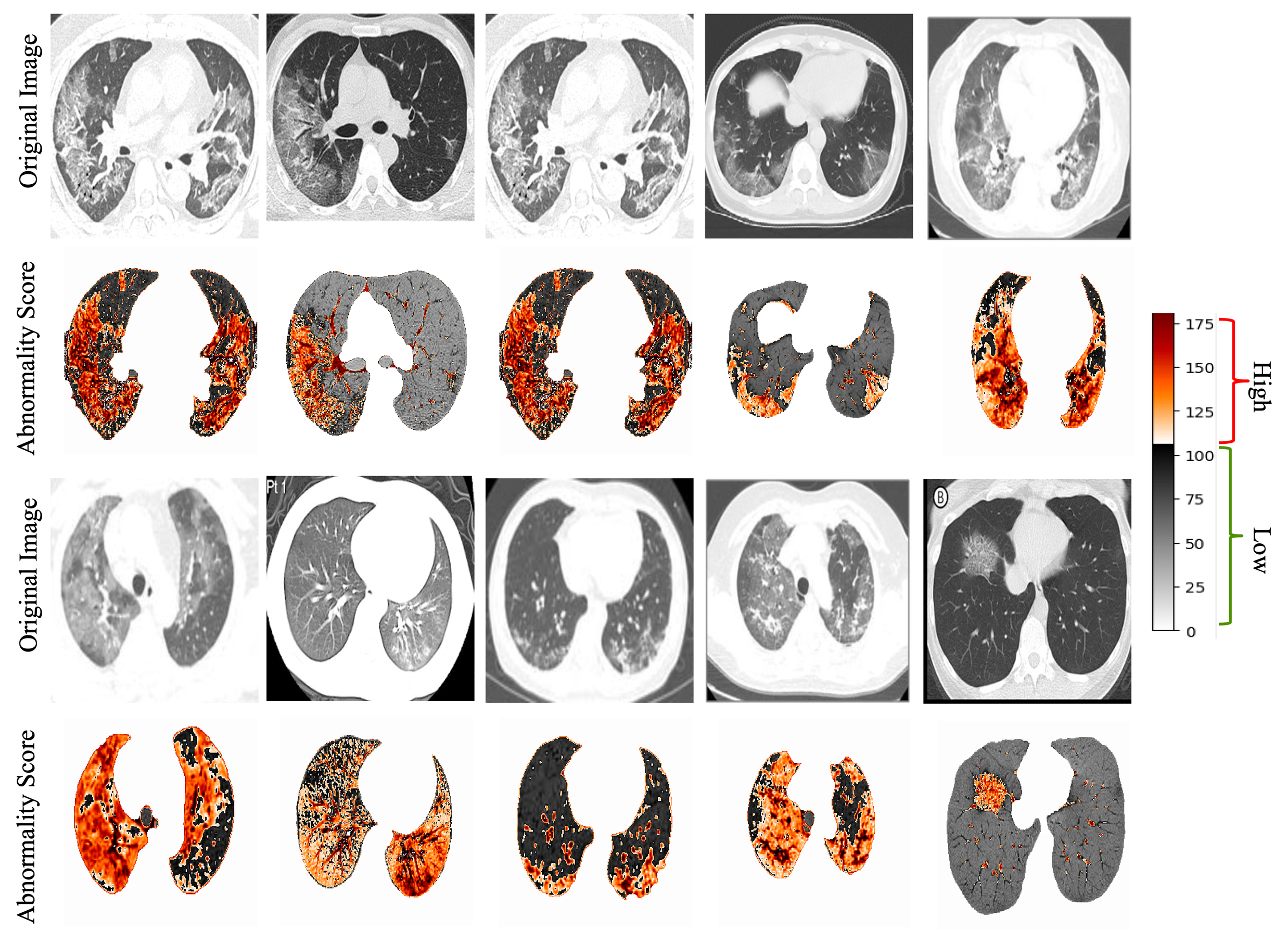

Supplement: S1 File — (ZIP) [file pone.0263916.s001.zip › Figure9.tif]
