# Supplementary material for: AI-driven quantification of ground glass opacities in lungs of COVID-19 patients using 3D computed tomography imaging
Source: PLoS One. 2022 Mar 14;17(3):e0263916. doi: 10.1371/journal.pone.0263916 (PMC8920286; doi:10.1371/journal.pone.0263916)
Supplement: S2 File — (PPTX) [file pone.0263916.s002.pptx]

## Slide 1
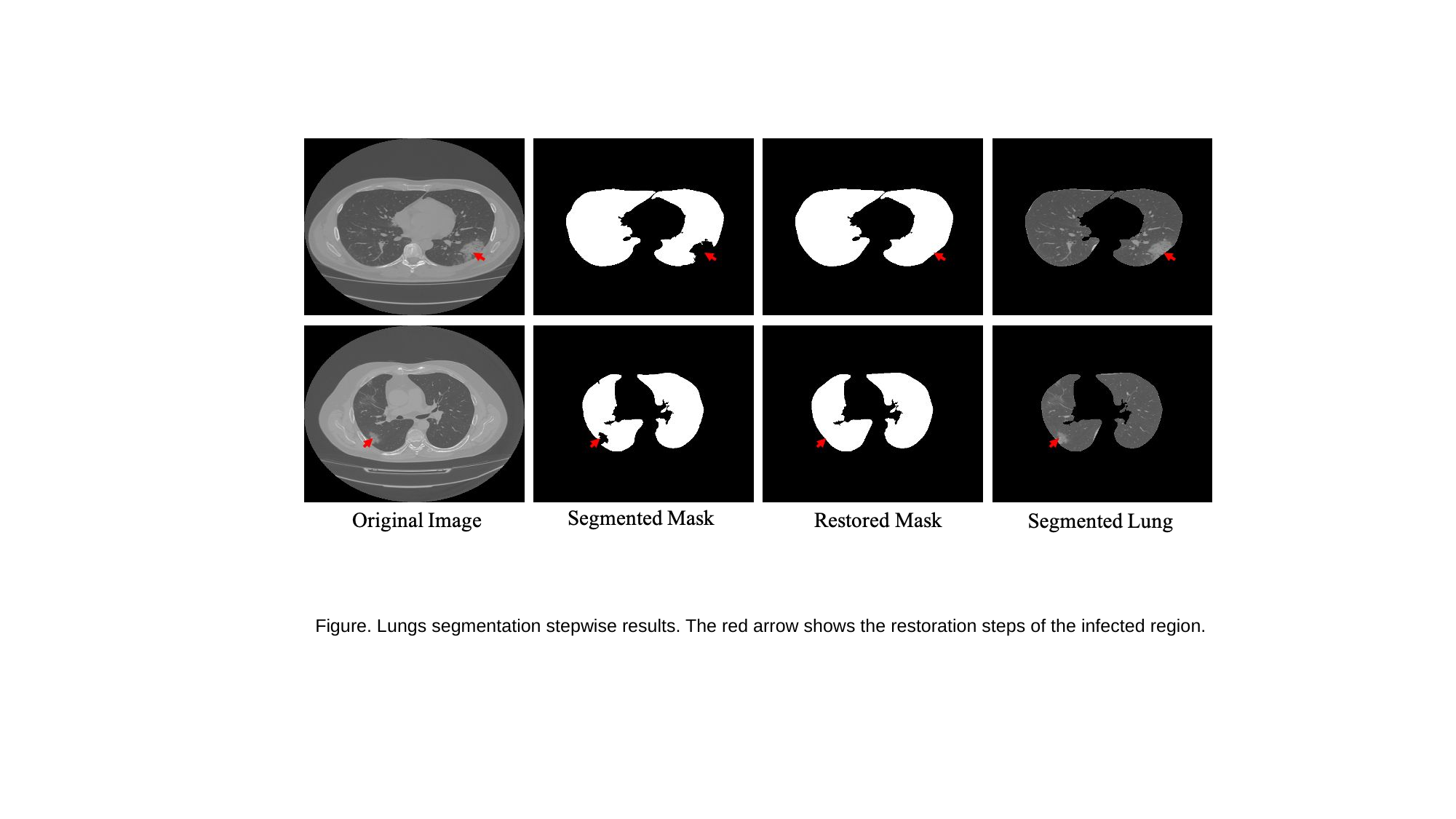

Figure. Lungs segmentation stepwise results. The red arrow shows the restoration steps of the infected region.

## Slide 2
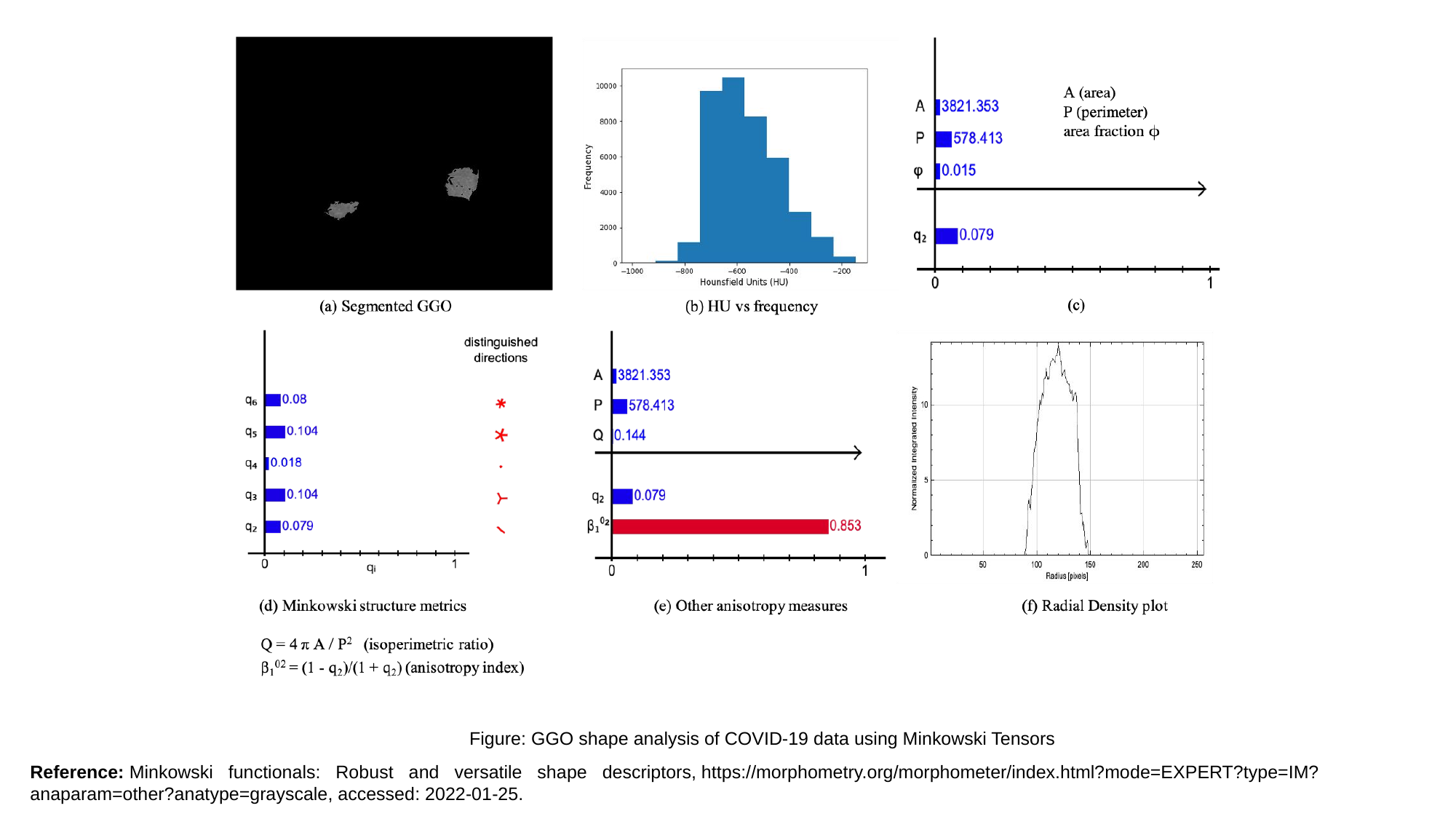

Figure: GGO shape analysis of COVID-19 data using Minkowski Tensors
Reference: Minkowski functionals: Robust and versatile shape descriptors, https://morphometry.org/morphometer/index.html?mode=EXPERT?type=IM?anaparam=other?anatype=grayscale, accessed: 2022-01-25.

## Slide 3
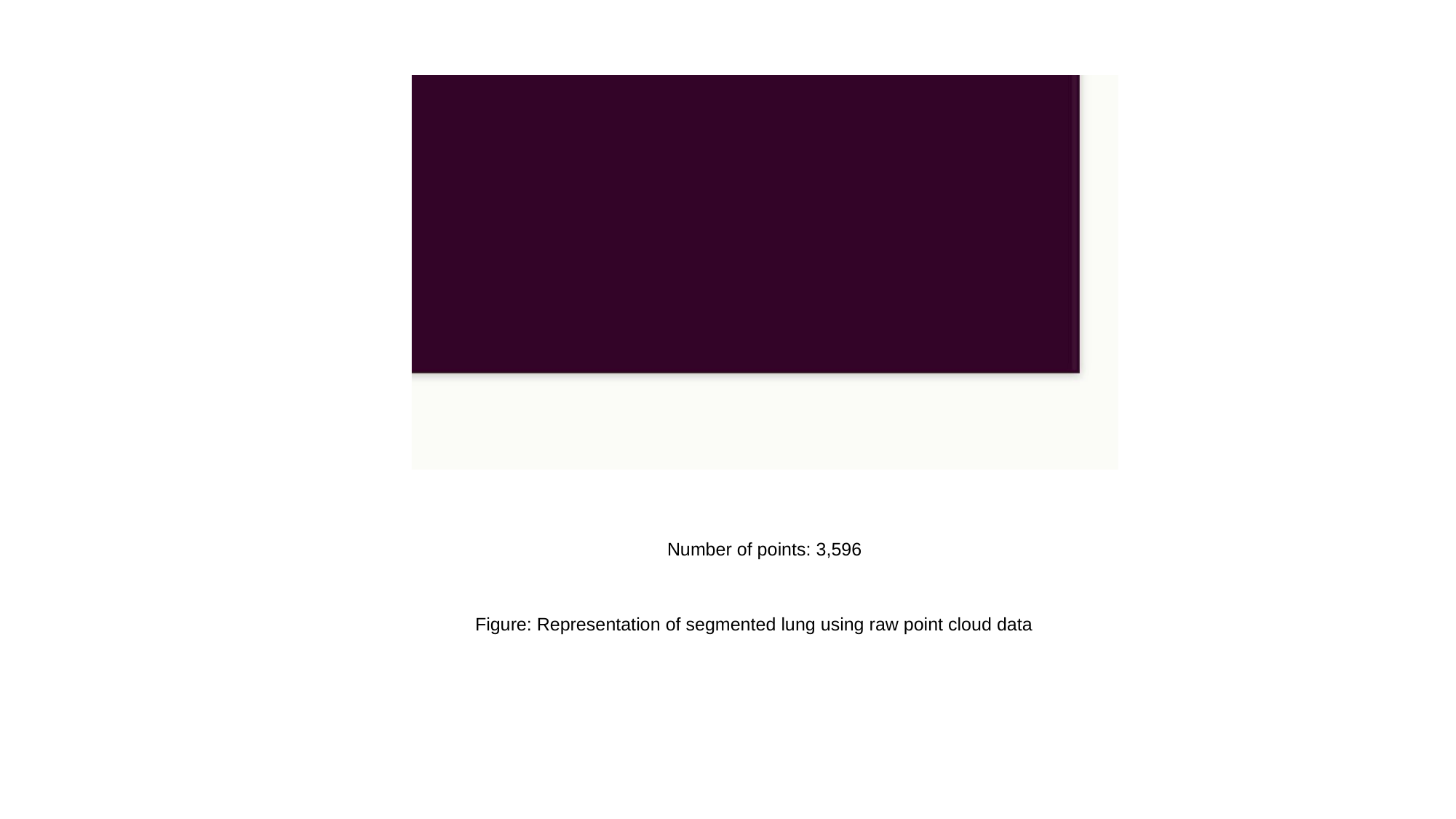

Number of points: 3,596
Figure: Representation of segmented lung using raw point cloud data

## Slide 4
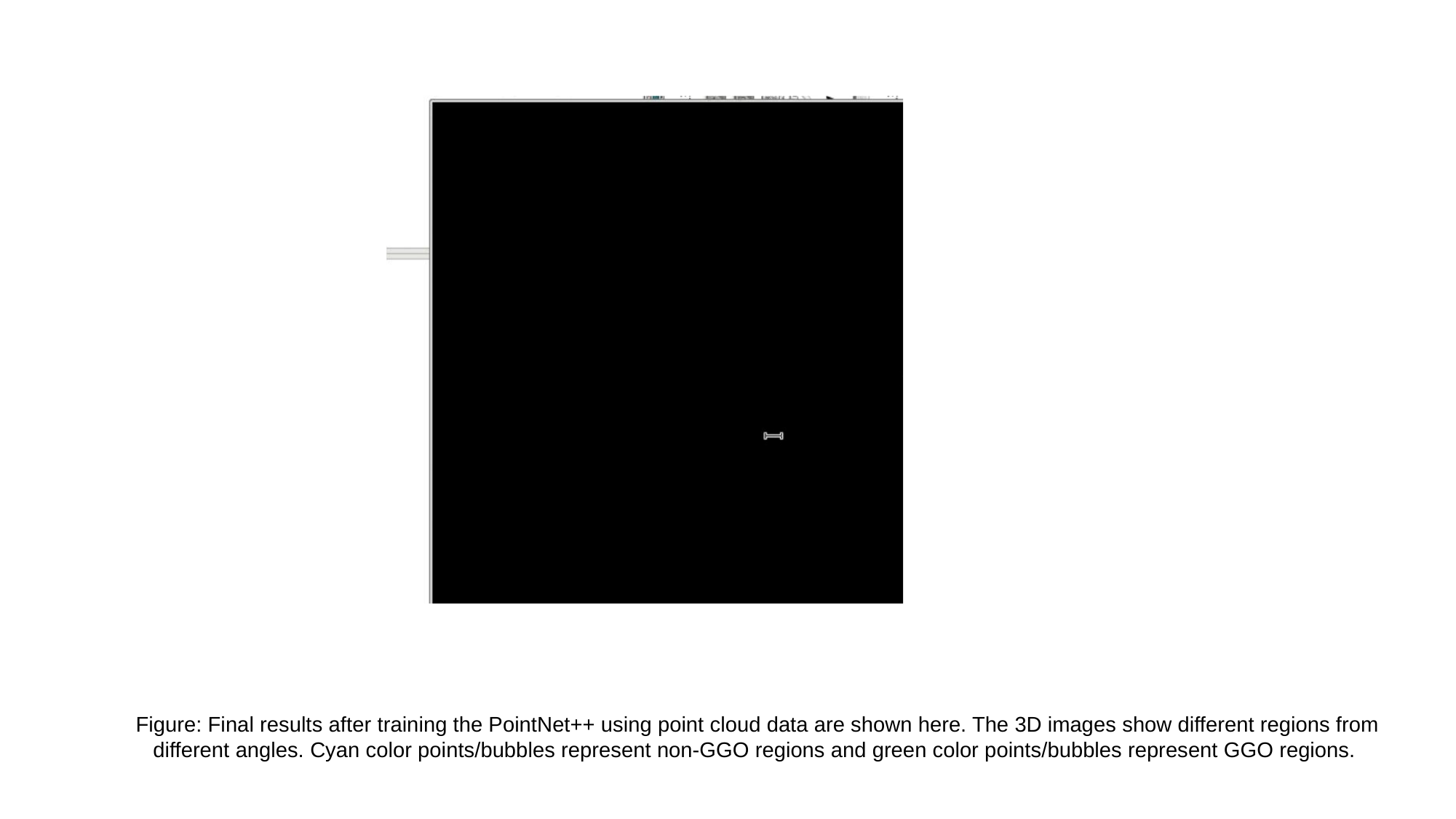

Figure: Final results after training the PointNet++ using point cloud data are shown here. The 3D images show different regions from different angles. Cyan color points/bubbles represent non-GGO regions and green color points/bubbles represent GGO regions.
