# Supplementary material for: AI-driven quantification of ground glass opacities in lungs of COVID-19 patients using 3D computed tomography imaging
Source: PLoS One. 2022 Mar 14;17(3):e0263916. doi: 10.1371/journal.pone.0263916 (PMC8920286; doi:10.1371/journal.pone.0263916)
Supplement: Attachment — Submitted filename: Response to Reviewers.docx [file pone.0263916.s003.docx]

**Response to Reviewers**

**Manuscript Number:** PONE-D-21-35862

**Manuscript Title:** AI-DRIVEN QUANTIFICATION OF GROUND GLASS OPACITIES IN LUNGS OF COVID-19 PATIENTS USING 3D COMPUTED TOMOGRAPHY IMAGING

***Reviewer #1: The proposed method is good. Manuscripts have been written with the standards of Scientific Writing Publications. Manuscript need equip with a recapitulation of test results for all tested data. References from old journal articles changed to the latest issue.***

**Response:** We appreciate the reviewer's insightful comments and ideas. We have uploaded the step-by-step results of our analysis to Google Drive. The below Google Drive link is available in the Supplementary Material.

[https://drive.google.com/drive/folders/1q8U5JesS6DgStYXohaGisl2y39rSS4RC?usp=sharing](https://urldefense.com/v3/__https:/nam11.safelinks.protection.outlook.com/?url=https*3A*2F*2Furldefense.com*2Fv3*2F__https*3A*2F*2Fnam11.safelinks.protection.outlook.com*2F*3Furl*3Dhttps*3A*2F*2Fdrive.google.com*2Fdrive*2Ffolders*2F1q8U5JesS6DgStYXohaGisl2y39rSS4RC*3Fusp*3Dsharing*26data*3D04*7C01*7Cashish.sharma*40emory.edu*7Cbf809fbe16a940baf9d208d93dcdb298*7Ce004fb9cb0a4424fbcd0322606d5df38*7C0*7C0*7C637608775978407245*7CUnknown*7CTWFpbGZsb3d8eyJWIjoiMC4wLjAwMDAiLCJQIjoiV2luMzIiLCJBTiI6Ik1haWwiLCJXVCI6Mn0*3D*7C1000*26sdata*3D6pYtI2CG5OChjn1PwKetmGfZyJJ1PV90oJhBKvx*2BwwE*3D*26reserved*3D0__*3BJSUlJSUlJSUlJSUlJSUlJSUlJSUlJQ!!LIr3w8kk_Xxm!4GwDGuDkjVa76vVxjSqeQA5S9SzHdo2_VzggowmqGK4Ic8wys_Vxs3_C75HRHg*24&data=04*7C01*7Cmonjoy.saha*40emory.edu*7C7f6925a6ec744e9def5808d940abcc2a*7Ce004fb9cb0a4424fbcd0322606d5df38*7C0*7C0*7C637611929028250207*7CUnknown*7CTWFpbGZsb3d8eyJWIjoiMC4wLjAwMDAiLCJQIjoiV2luMzIiLCJBTiI6Ik1haWwiLCJXVCI6Mn0*3D*7C1000&sdata=y3iCXcm2twwM17hsSDlw8Ck202J5mimgMlzKyUiIyGA*3D&reserved=0__;JSUlJSUlJSUlJSUqKioqKioqKiUlKioqKioqKioqKioqJSUqKiUlJSUlJSUlJSUlJSUlJSUl!!LIr3w8kk_Xxm!-eGcTnW2T0Te92aJAxmDe0XK5ftA-nLC9FD4z3o12p7ITGtTGeNg3voWTyDY2Q$)

For the original dataset, please check the Materials and methods of the manuscript. We are unable to upload original data as redistribution of original data is not allowed. We have also added one "**Additional_results.pptx**" file as a Supplementary Material, which contains animated videos and additional results.

As suggested by the reviewer, we also have updated the below sentence in our revised manuscript and accordingly updated the references. All the changes have been highlighted using yellow color.

***Main Manuscript [page 3]***

It has infected over 342 million people and killed over 5.5 million people at the time of writing this paper (source: <https://coronavirus.jhu.edu/map.html>).

**References of Supplementary Material**:

[1] Stare J, Maucort-Boulch D. Odds ratio, hazard ratio and relative risk. Metodoloski zvezki. 2016;13(1):59.

**Additional_results.pptx.** This file contains some additional results

***Reviewer #2:***

***Comment: The author proposed a manuscript which has a title "AI-DRIVEN QUANTIFICATION OF GROUND GLASS OPACITIES IN LUNGS OF COVID-19 PATIENTS USING 3D COMPUTED TOMOGRAPHY IMAGING". This research come up with a hot issue nowadays. The manuscript is explained structurally.***

**Response**: The authors are thankful to the reviewer for their constructive and inspirational comments. We have addressed all the comments raised by the reviewer. Changes have been highlighted in the revised manuscript using yellow color.

***Q1. in line 252, author mentions that the network is the same as VGG-16 but author reduce the first four convolution layers into two. It was done to achieve best performance. how author proof this one?***

**Response:** Thank you so much for pointing out this question. Please find our response below. The below sentences have been updated in the revised manuscript. The changes have been highlighted using yellow color [page 13].

The first four convolution layers of the VGG-16 network accept images of size 224x224 pixels and 112x112pixels. Our input data size is 512x512pixels. To accommodate our images into VGG-16, we had to resize the image. But resizing large images into smaller sizes at the initial stage has a chance of information loss. Hence, we have replaced the four convolution layers with two layers. Accordingly, we have changed the kernel size and max-pooling operations. Moreover, in the VGG-16 network, the final layer is the fully connected layer. In our case, the final layer is the Cox layer, which computes abnormality scores. If we use exactly the VGG-16 network, we will not be able to compute abnormality scores.

***Q2. author mention that, the MosMedData is used in this research. what is the image size? because if author modified model from VGG-16 which has its on pixel size? what kind of preprocessing was done to the dataset?***

**Response:** Thank you so much for asking this question. The image size of MosMed data is 512 x 512 pixels. We have modified the first four convolution layers of the VGG-16 network to accommodate the 512 x 512 pixels images. Accordingly, we have changed kernel size and max-pooling operations. We have updated this information in the revised manuscript.
